# Potential of Fenofibric Acid as Topical Eye Drops for Management of Dry Eye Syndrome

**DOI:** 10.3390/pharmaceutics18080998

**Published:** 2026-08-13

**Authors:** Guilin Tan, Miao Chen, Peng Xie, Chengying Bian, Weizhuo Wang, Shaoqun Wu, Yuanhui Jin, Lingyun Cheng

**Affiliations:** 1 Eye Hospital and School of Ophthalmology and Optometry, Wenzhou Medical University, 270 Xueyuan Road, Wenzhou 325027, China; guilintan@hotmail.com (G.T.); chenmiaow@outlook.com (M.C.); xiepengyk@foxmail.com (P.X.); bianchengying@foxmail.com (C.B.); weizhuochn@163.com (W.W.); 18327053323@163.com (S.W.); yuanhuij@outlook.com (Y.J.); 2 Guizhou Provincial People’s Hospital, Guiyang 550002, China; 3 Department of Ophthalmology, Jacob’s Retina Center at Shiley Eye Institute, University of California San Diego, La Jolla, CA 92093, USA

**Keywords:** fenofibric acid (FFA), ocular safety and toxicity, ocular pharmacokinetics of FFA, FFA goblet cells protection, benzalkonium chloride-induced dry eye model, Sprague–Dawley rat

## Abstract

**Background/Objectives:** Ocular surface inflammation has been identified as a key causative factor for dry eye. The current study investigates the feasibility, safety, and efficacy of fenofibric acid (FFA) as a topical eye drops in controlling ocular surface inflammation. **Methods:** FFA was tested for its dissolution profile and its cytotoxicity in vitro, and its permeability through ocular surface tissues was tested ex vivo. A safe dose was tested on the Sprague–Dawley (SD) rat eye as an eyedrop for ocular safety and ocular pharmacokinetics. The efficacy was tested on a benzalkonium chloride (BAC)-induced dry eye model in the SD rats. The outcome measurements were analyzed against the untouched contralateral eyes of the animals. **Results:** The study found that FFA had a similar dissolution profile in PBS (phosphate-buffered saline) as is in saline; however, the saturated concentration in PBS was 81 times higher. FFA had an IC50 (half maximal inhibitory concentration) of 282.4 µM on HCECs (human corneal epithelium cells). The FFA permeation rate for the cornea was 1.77 × 10^−6^ µg/cm^2^/min, 4.95 × 10^−6^ µg/cm^2^/min for the conjunctiva, and 11.32 × 10^−6^ µg/cm^2^/min for the complex of the sclera/choroid. A regimen of twice-a-day eyedrops (500 µg/mL) demonstrated good ocular safety and therapeutic efficacy on the BAC-induced dry eye model in SD rats, with significant effects on the reduction in corneal edema and on preventing the loss of goblet cells from dry eye pathology. **Conclusions:** These findings strongly suggest that FFA eye drops at a concentration of 500 µg/mL may effectively control ocular surface inflammation and relieve dry-eye discomfort.

## 1. Introduction

Fenofibrate is a medication to control high levels of cholesterol and triglycerides in the blood. In the two randomized clinical trials [1,2] (FIELD, the Fenofibrate Intervention and Event Lowering in Diabetes; and ACCORD, Action to Control Cardiovascular Risk in Diabetes), the oral fenofibrate used for control hyperlipidemia retarded diabetic retinopathy progression. In the FLELD study, the significant low prevalence of macular edema and fewer demands for laser coagulation were found not to be associated with lowered plasma lipids, which suggests there may be other biological effects from fenofibrate to retard diabetic retinopathy [1]. Recent in vitro studies have demonstrated that fenofibrate and its active metabolite (fenofibric acid, FFA) suppress microvascular endothelial inflammation and apoptosis through adenosine-monophosphate-activated protein kinase activation [3]. In a rat retinal endothelial cell study, FFA also markedly inhibited the high-glucose-induced overexpression of Cox-2 (cyclooxygenase-2), a critical mediator of inflammation in the extracellular matrix [4]. Cox-2 is known to contribute to the increased microvascular permeability seen in diabetic retinopathy [4]. The in vitro and in vivo studies suggest that FFA may have directly inhibited retinal inflammation and angiogenesis as an agonist of PPARα (the peroxisome-proliferator-activated receptor type α) [5,6]. These experimental pharmacological findings [7] have prompted the investigators to look into the intravitreal or topical application of the fenofibrate on animal models of diabetic retinopathy [6,8]. In contrast to the systemic usage, ocular application may have the advantages of achieving better ocular drug availability and reducing the risks of systemic side effects such as abnormal kidney function tests [9] or acute pancreatitis or pulmonary emboli [1].

Currently, XIIDRA (lifitegrast) is the only uniquely designed therapeutics to target lymphocyte-function-associated antigen 1 (LFA-1) to block its synapse on ICAM-1 on the ocular surface. Lifitegrast can reduce T-cell-mediated ocular surface inflammation; however, dry eye is a multifactorial disease, and further targeting the other inflammatory pathway would enhance the treatment efficacy and elevate patient care. More recent studies have suggested that the PPAR (Peroxisome-Proliferator-Activated Receptor) signaling pathway is involved with ocular surface inflammation [10,11,12]. FFA is a potent PPAR_alpha_ agonist. More evidence has emerged that FFA can downregulate the inflammatory cytokines such as IL-6, TNF-α, and IL-17 via stimulating PPAR_alpha_ receptors [10,13,14]. These cytokines (such as MMP-9, IL-1β, IL-6, TNF-α, MIP-2, and ICAM-1) are key contributors to the ocular surface inflammation in dry eye disease [15]. We hypothesize that topical fenofibric acid (FFA) could be an effective alternative therapy for the management of dry eye disease. The current study started from profiling the FFA dissolution and cytotoxicity in vitro, and then determined the FFA penetration of ocular surface tissues in ex vivo, and finally moved to preclinical studies to assess FFA ocular safety, ocular pharmacokinetics, and the therapeutic potential as a topical application on a rat dry eye model.

## 2. Materials and Methods

### 2.1. Study Design

The research followed the ethical guidelines and the Statement for the Use of Animals in Ophthalmic and Vision Research set by the Association for Research in Vision and Ophthalmology. Fenofibric acid (FFA) is the active phase-I metabolite of fenofibrate and has stronger anti-inflammatory power than the fenofibrate itself [16]. Therefore, the current study used FFA as an active pharmaceutical ingredient (API) to investigate its safety and potential as a topical application for ocular surface inflammation (dry eye). The rat eye may not be an ideal animal model of posterior segmental eye diseases for testing a topical drug delivery method, due to the short globe axis and proportionally large cornea-to-sclera ratio. However, it is considered a suitable model for testing topical eye drug application on anterior segmental eye diseases.

### 2.2. Investigation of Solubility and Dissolution Profile of Fenofibric Acid

The fenofibric acid and fenofibric acid standard (purity 99.94%, molecular weight 318.76 g/mol) were purchased from MedChemexpress (MCE, Monmouth Junction, NJ, USA). Phosphate-buffered saline (PBS) powder was purchased from Ding Guo Prosperous Incorporated (Beijing Dingguo Changsheng Biotechnology Co., Ltd., Address: Room 606, Mudan Office Building, Yard B9, Huayuan East Road, Haidian District, Beijing, China). PBS solution was prepared from the powder and deionized water with sonication and filtration (0.22 µm) to produce a 0.01 M solution before titrating pH to 7.4. Fenofibric acid powder 450 mg was weighted into a dialysis bag with a molecular weight cut-off of 14,000 Da (Biosharp, Hefei, China). The dialysis bag was placed into a BD tube either with 45 mL of PBS or 0.9% sodium chloride saline (quadruplicate for testing in each solution). The sample tubes were placed on a shaker at 37 °C with the speed of 100 rpm. Then, a 200 µL sample was taken from the tube at time points of 0.5 h, 1 h, 3 h, 5 h, 7 h, 24 h, 48 h, 72 h, 96 h, 120 h, and 144 h. After the sample was taken at each time point, a fresh 200 µL PBS was added back into the tube to restore the dissolution volume. The simulated tear fluid was not used for this study due to the better stability of PBS and the consistency for the subsequent quantitation by Ultra-Performance Liquid Chromatography (UPLC, Shimadzu, Japan).

The concentration of fenofibric acid samples was determined by the UPLC. The mobile phase consisted of ultrapure water and methyl alcohol under isocratic conditions at a flow rate of 0.3 mL/min. The injection volume was 10 µL, the detecting light wave length was 286 nm, and the run time was 8 min.

### 2.3. Cytotoxicity Assay In Vitro

The current study was undertaken to evaluate the potential of an ocular topical application of fenofibric acid on ocular surface inflammation. Cornea epithelium cells have high turnover rate to maintain health surface of the cornea. In the current study, human cornea epithelium cell (HCEC) line (obtained from the American Type Culture Collection, Manassas, VA, USA) was used to determine the margin of safety. The saturated fenofibric acid concentration was used to make step-down diluted concentrations so that the resultant testing concentrations de-escalated from 488 µg/mL to 0.003 µg/mL at a half-log scale. The FFA solution consists of only FFA and PBS, no other solubilizers or preservatives. The HCEC was cultured in DMEM/F12 medium with 10% FBS and 5 µg/mL insulin and antibiotics/antifungals. A 96-well plate was used and each well was seeded with 7000 cells to start with. When the cells were well attached to the well wall 24 h later, the culture medium was exchanged with the prepared culture medium containing 25% volume of testing solution (higher FFA concentrations were prepared in PBS and lower FFA concentrations in saline). Each testing concentration had 10 well replicates along with PBS controls (no fenofibric acid) or saline control (no fenofibric acid). After 48 h of culture, the one containing 10% CCK-8 (Biosharp, Life Sciences, Hefei, China) medium was exchanged for another two hours of culture, before optical density (OD) of each well was read out at 450 nm absorbance under a microplate reader (SpectraMax M5, Molecular Devices, Shanghai, China).

### 2.4. Permeability Through the Ocular Tissues in Ex Vivo

**Figure 1:**
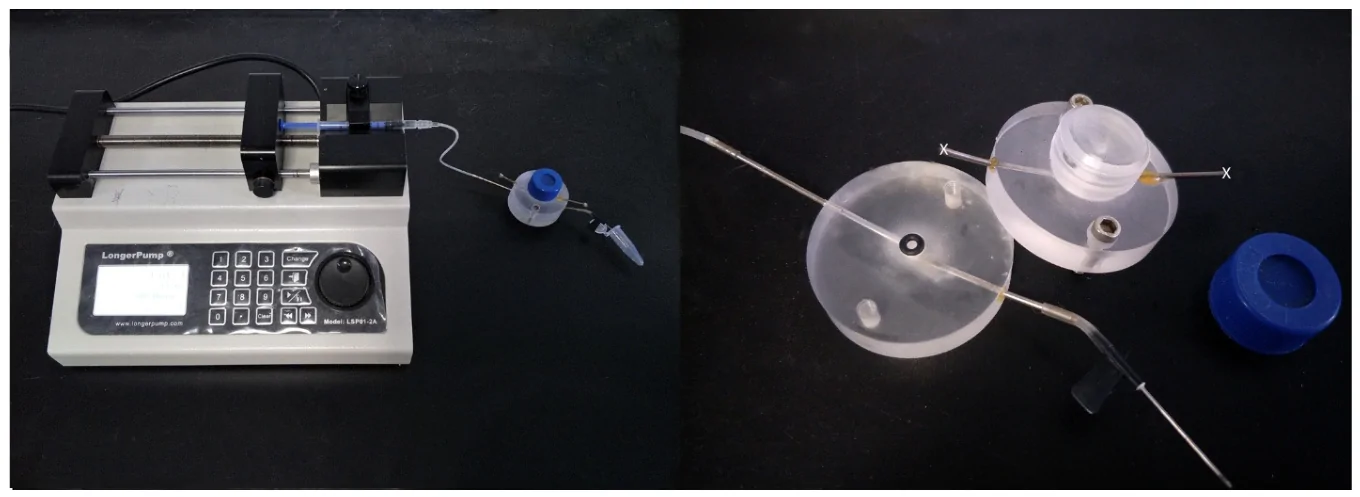
The left panel shows the setting of the drug permeation experiment with the permeation device, fluid lines, irrigating syringe, and the syringe pump. The right panel shows a disassembled permeation device consisting of the lower part and the upper part. The lower part has the irrigation inlet tubing for irrigation and the outlet tubing for sample collection. For the upper part, the top port is for drug solution loading and can be airtight sealed with the blue cap. The inlet and outlet of the upper chamber were sealed for this study.

Four SD rats (8 eyes) were used for this study. All animal usage was in accordance to the ARVO statement for the use of animals in ophthalmic and vision research. There were eight samples for conjunctiva or cornea and 16 samples for sclera/choroid. For each eye, conjunctiva was sampled first, starting from the eye lid margin and ending at the limbus to ensure it was large enough to mount to the permeation chamber. After the globe was enucleated, the cornea was excised along the limbus while the remaining eye cup was divided into two halves. The eye cup content, including the retina, was carefully removed to harvest clean sclera/choroid. A 6 mm disposable biopsy punch was used to sample discs of cornea, conjunctiva, and sclera. The sample disc was loaded onto a customer-made Ussing chamber device, which consists of upper and lower parts with a 3 mm orifice, as shown in Figure 1. The testing sample was placed on the O-ring with the external part facing the donor compartment, and the two parts were secured by two screws to prevent leaking. The FFA solution of 40 µL (500 µg/mL) was loaded into the upper chamber via the top port while the inlet and outlet of the upper chamber were blocked for this study purpose. The lower chamber was irrigated by a 1 mL syringe controlled by a syringe pump at a flow rate of 2 µL/min; the fluid from the outlet of the lower chamber was guided via tubing into a collecting vial. The sample was collected at 30 min, 60 min, 90 min, 120 min, 150 min, and 180 min. Sample volume at each time point had 60 µL that was subjected to UPLC to determine the concentration of fenofibric acid. The tissue permeability of fenofibric acid was calculated using the formula below, and the unit of Pc is cm/sec [17,18].

Pc = (C_180min_ − C_30min_) × V/AtC_0_

C_180min_ = concentration of fenofibric acid in the sample collected at the 180 min time point. Similarly, C_30min_ = concentration of fenofibric acid in the sample collected at the 30 min time point; V = fluid volume in the lower chamber, which was 60 µL; A = the effective diffusion area (1.5 by 1.5 mm × 3.14); t = the time interval between the samplings; and C_0_ = the FFA concentration in the upper chamber. x = multiplied by.

### 2.5. Ocular Safety Evaluation Following Topical Administration

Two FFA concentrations were tested on 8 Sprague–Dawley rats for 2 weeks. High concentration was 5 times the concentration of IC_50_ that was determined from the cytotoxicity experiment, and low concentration was the concentration of IC_50_. The FFA solution was tested for pH values at room temperature by a precision pH test strip (Shanghai Sanais Reagent Co., Ltd., Shanghai, China). The rats were weighed for a starting body weight before the starting. Only one eye of each rat was used for the eye drop installation, while the contralateral eye was used as a normal control for safety evaluation. The testing solution was installed twice per day, one drop each time, and the rat’s head was restrained for 30 s after each installation to prevent blinking or head shaking. The anterior segment of the rat eyes was examined by a biomicroscope (slit lamp, Carl Zeiss Meditec, Jena, Germany) for a 0–3 grading of conjunctiva congestion and cornea fluorescence staining on day 1, day 3, day 7, and day 14. Electroretinogram (ERG) was performed on both eyes on day 7 and day 14. Body weight was again measured on the same scale prior to the animal sacrifice. The eyeballs were enucleated, and the ocular tissues separated for quantitation of fenofibric acid from each type of ocular tissue using UPLC. For the ERG procedure, the pupils were dilated with 0.5% tropicamide and 0.5% phenylephrine eye drops (Santen Pharmaceutical Co., Ltd., Osaka, Japan) and dark-adapted for 2 h. Under a dim red light, after sedation with 1 mL of 10% chloral hydrate (per 300 g of body weight), a 2.5% methylcellulose gel was applied to each eye after 0.01% propantheline topical anesthesia, and a gold 4 mm loop electrode was placed on the cornea to record the ERGs. Reference needles were inserted into two cheeks, and a ground electrode was inserted into the tail, respectively. A full-field ERG was recorded simultaneously from both eyes. All stimuli were presented in a Ganzfeld dome (Roland Q400; Wiesbaden, Germany). Dark-adapted ERG was elicited with the flashlight intensity of 0.01 and 3 cd·s/m^2^. Five flashes were averaged. After dark-adapted ERG, animals were light-adapted for 5 min under the light intensity of 25 cd/m^2^ and single-flash photopic ERGs with the flash light intensity of 3 cd·s/m^2^ were recorded. Five flashes were averaged. Finally, 30 Hz flicker ERGs were recorded with the same light intensity as the photopic ERGs against the background light of 25 cd/m^2^.

### 2.6. Ocular Pharmacokinetics Following a Topical Application

To investigate ocular concentration changes over time, FFA concentrations in the cornea and conjunctiva were quantitated after one drop (25 µL) of fenofibric acid (500 µg/mL). For this study, 28 male SD rats were randomly allocated into 7 groups, 4 animals in each group. Only the right eye of each animal was used for the study, and the contralateral eye was not touched. After one drop of fenofibric acid, four animals at one of 7 time points (15 min, 30 min, 1 h, 2 h, 3 h, 5 h, and 7 h) were sacrificed to collect the conjunctiva and cornea from both eye globes to quantitate the fenofibric acid in these tissues. To avoid cross-contamination, the snap-frozen technique was used, as we previously reported [19,20]. Before the sacrifice, 2 mL of blood was sampled from the heart for plasma extraction and fenofibric acid quantitation.

### 2.7. Design of Animal Study for Efficacy Evaluation

**Figure 2:**
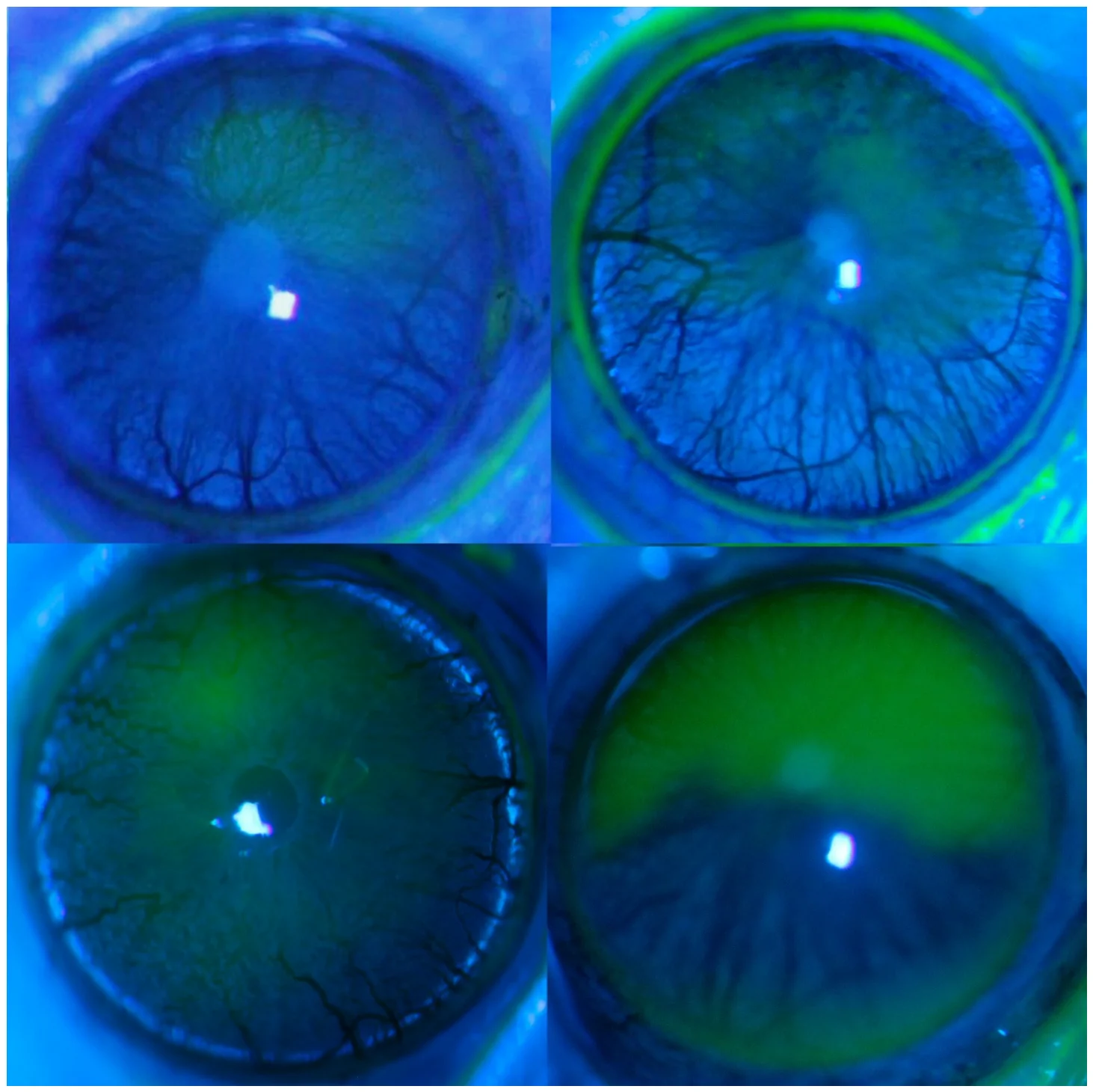
Fluorescein cornea staining scheme for grading. The upper left panel demonstrates a confluent superficial staining covering less than one quadrant, graded as 1; the upper right panel demonstrates a general confluent superficial staining covering roughly two quadrants, graded as 2; the lower left panel demonstrates a confluent superficial staining within roughly three quadrants and an area of deeper than superficial within one quadrant, graded as 5 (3 × 1 + 2); and the lower right panel demonstrating deep staining covering roughly two quadrants of the cornea, graded as 6 (3 + 3).

Fifteen male SD rats were used, with the right eye as the study eye and the fellow eye untouched. In this pilot study, we used only male rats in consideration of removing female hormonal fluctuations that may confound the dry eye uniformity. Before entering the study, all rats had anterior eye exams with a slit lamp and no clinically observable abnormality on the ocular surface. All animal handlings are in compliance with the ARVO Statement for the Use of Animals in Ophthalmic and Vision Research. For this dry eye model study, rats were randomly assigned into the FFA intervention (9 rats) or saline (NS) intervention (6 rats) group at a 3-to-2 ratio. Firstly, 0.2% benzalkonium chloride (BAC) was instilled 3 times daily (8 am, 3 pm, and 10 pm) with daily corneal fluorescein staining exams under a slit-lamp biomicroscope. When the cornea demonstrated Grade-2 staining reaching 50% of the cornea or revealed dense staining plaque over 25% of a cornea (Figure 2), BAC induction eye drops were stopped and FFA or NS intervention were initiated. FFA or NS drops twice a day (10 am, and 4 pm) were performed for 28 consecutive days. Before the start of the intervention and during the intervention, cornea staining, cornea edema, and limbus neovascularization were graded under a slit-lamp biomicroscope on day 4 or day 7, day 14, day 21, and day 28. Goblet cell impression cytology was performed each week after the starting of intervention.

1. Grading of cornea fluorescein staining: The cornea was divided into 4 quadrants. Grade-1 was graded as confluent superficial staining with a view of the underneath iris vessels; Grade-2 as confluent staining obscuring the view of underneath iris vessels; and Grade-3 as deep staining blocking the view of the underneath iris vessels. The highest grade for a cornea is 12.
2. Grading of cornea edema: The cornea edema was graded for both severity and area. The sum of severity and area was used to quantitate cornea edema. For the severity, Grade-1 is the edema under which iris vessels less clear; Grade-2 is the edema obscuring the underneath iris vessels; Grade-3 is the edema almost blocking view of the underneath iris vessels; and Grade-4 is the opaque edema with surface bulging and bumping. For the area grading, Grade-1 = fluorescein staining area less than 25% of the cornea; Grade-2 = the fluorescein staining area between 25 and 50% of the cornea area; Grade-3 = the fluorescein staining area between 50 and 75% of the cornea area; and Grade-4 = the staining over 75% of the cornea area. The highest grade for a cornea is 16.
3. Grading of cornea neovascularization: The cornea was divided into 4 quadrants. Each quadrant was graded, and maximum score is 16. Grade-1: new vessels stretch from the limbus into the clear cornea but cover less than 20% of a quadrant; Grade-2: new vessels cover less than 50% of a quadrant; Grade-3: the new vessels cover more than 50% but less than 75% the area of a quadrant; and Grade-4: over 75%.
4. Conjunctival impression cytology: The procedure was performed after one drop of proparacaine eye drops. A prepared 2 mm filter paper disc was used to contact the conjunctiva 2 mm away from the limbus for 10 s. Then, the disc was placed into 95% ethanol within an EP vial under 4 °C for 10 min before periodic acid–Schiff (PAS) staining. Each eye was sampled at two locations, between 10 and 11 o’clock as well as between 1 and 2 o’clock. After the staining, the sample was sealed with a coverslip for microscopy. Under a light microscope, 4 areas were randomly selected under 10× magnification and photographed at 20×. ImageJ 1.53j (https://imagej.net/ij, accessed on 8 August 2026) was used to count the goblet cells.

At the end of the experiment, rats were sacrificed and eye globes enucleated for fixation (mixture of 10 mL formalin, 10 mL glacial acetic acid, and 80 mL of 80% ethanol).

### 2.8. Fenofibric Acid Determination

Ultra-performance liquid chromatography (UPLC/MS/MS) was used to determine the FFA concentration. The standard fenofibric acid was step-diluted into 0.1 ng/mL, 0.2 ng/mL, 0.5 ng/mL, 1 ng/mL, 2 ng/mL, 5 ng/mL, 10 ng/mL, 20 ng/mL, 50 ng/mL, and 100 ng/mL for standard curve construction. Each step of concentration had 3 replicates. The standard curve was used to determine the FFA concentration from in vitro and ex vivo samples.

For in vivo study samples, diclofenac acid was used as the internal standard. For aqueous humor and vitreous humor, a 20 µL sample was mixed with 20 µL acetonitrile and 20 µL internal standard acetonitrile solution (100 ng/mL). After 1 min vortex and 10 min centrifugation (14,000 rpm), a 50 µL supernatant was taken for the analysis. For the blood sample, 200 µL plasma was mixed with 20 µL acetonitrile containing the internal standard (100 ng/mL) and 200 µL 1 mol/L Na_2_CO_3_. Then, vortex the mixture with 3 mL of the ethyl acetate for 1 min before 10 min centrifugation (4000 rpm), taking the organic upper layer (2.7 mL) to dry down under N_2_ within a water bath, followed by reconstitution with 100 µL mobile phase (CAN: H_2_O, 1:1), and then centrifugation and filtration (96-hole filtration plate at 4 °C) for 5 min. The filtrate was subject to the analysis. For the cornea, conjunctiva, or sclera/choroid, 100 µL ammonium acetate and 400 µL acetonitrile were added into the weighted tissue in a 1.5 mL EP tube for overnight at 4 °C. The tissue was homogenized and moved into a 15 mL BD tube for further addition of the fenofibric acid internal standard and processed as described above for the plasma samples. The dry-down samples were reconstituted with 100 μL of mobile phase (vortex for 20 s) and filtered by centrifugation at 4000 rpm for 5 min (96-well filter plate, 4 °C). The filtrates were subjected to UPLC/MS/MS.

The column was Shim-pack XR-ODS III C18 (150 mm by 2 mm, 2.2 µm). The mobile phase was methanol-0.1% formic acid (80:20, *v*/*v*), the flow rate was 0.4 mL/min, the column temperature was 35 °C, and the loading sample volume was 10 µL. The lower limit of quantification was 1 ng/mL or 1 ng/g.

### 2.9. Statistical Analysis

For ex vivo permeation data, the nonparametric Wilcoxon each-pair comparison was used for comparing the three types of ocular tissues. For ERG data, the peak-to-peak amplitude was used as an outcome measurement. Day 7 and day 14 ERG data were pooled. A generalized linear mixed model was used to compare the dosing groups while adjusting for ERG time points. A post hoc test was performed by running Dunnett. For the scores of corneal edema, fluorescent staining, and neovascularization, a reduction score was derived from subtracting the score at the beginning of the intervention (day 0) by the score at each follow-up time point. Such a subtraction generated a number for each time point, which was used as repeated measures for the statistics. A generalized linear mixed model was used to compare the reduction scores between FFA and saline interventions while using rat ID as a random effect in the regression model. For the conjunctival impression cytology, it was performed at two locations in each eye. A generalized linear mixed model was used to compare the number of goblet cells among the intervention groups. All statistics were conducted within the SAS JMP platform (version 18).

## 3. Results

### 3.1. Dissolution and Solubility

**Figure 3:**
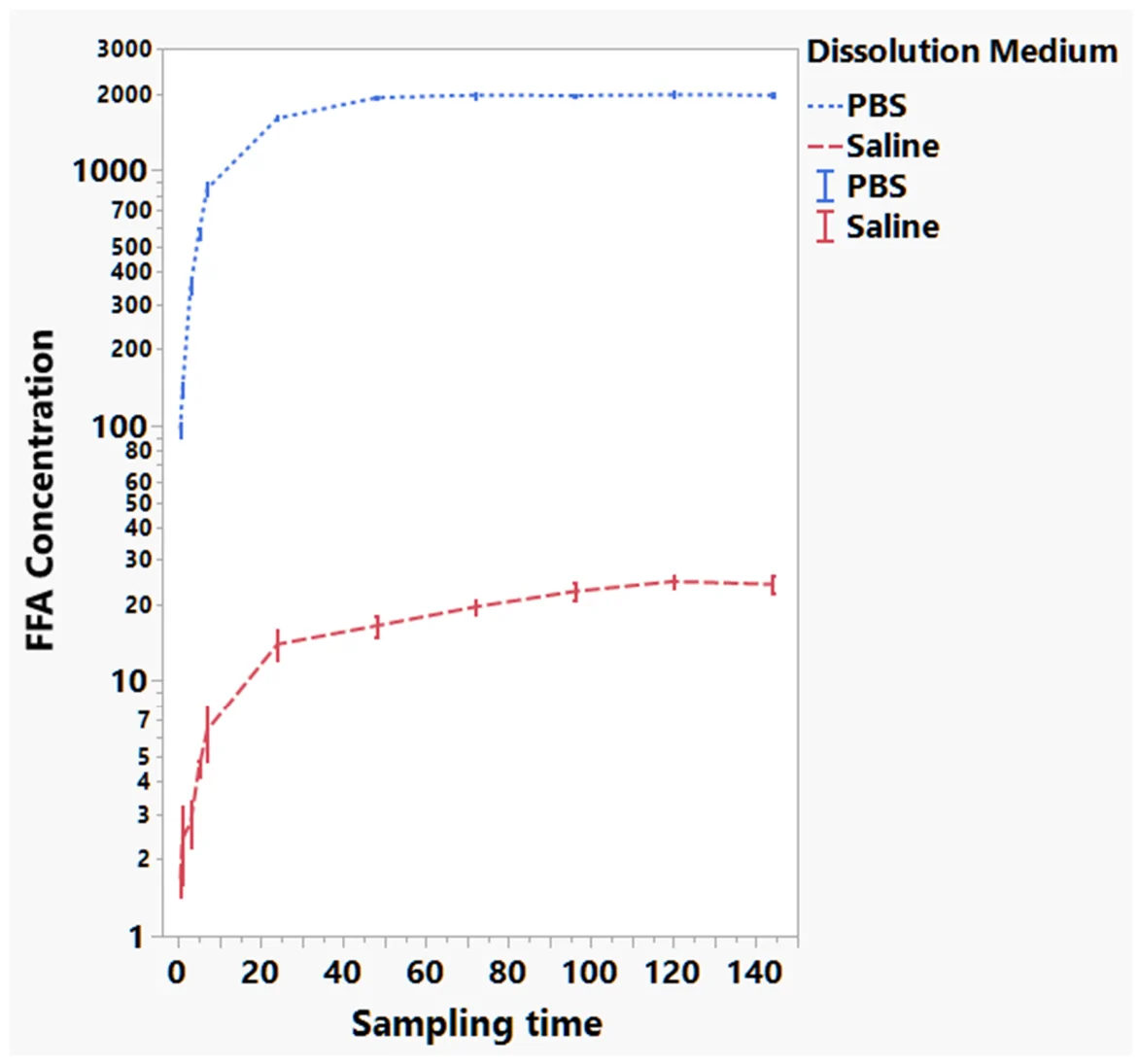
FFA release profiles and solubility in PBS or saline under sink condition.

The FFA concentration in PBS reached saturation within 72 h, while it takes 120 h in saline to saturate. The saturated concentration in saline was 24.2 ± 3.2 µg/mL but was 1954.68.1 ± 59.19 µg/mL in PBS. The dissolution profiles are presented in Figure 3.

### 3.2. In Vitro Cytotoxicity Assay

**Figure 4:**
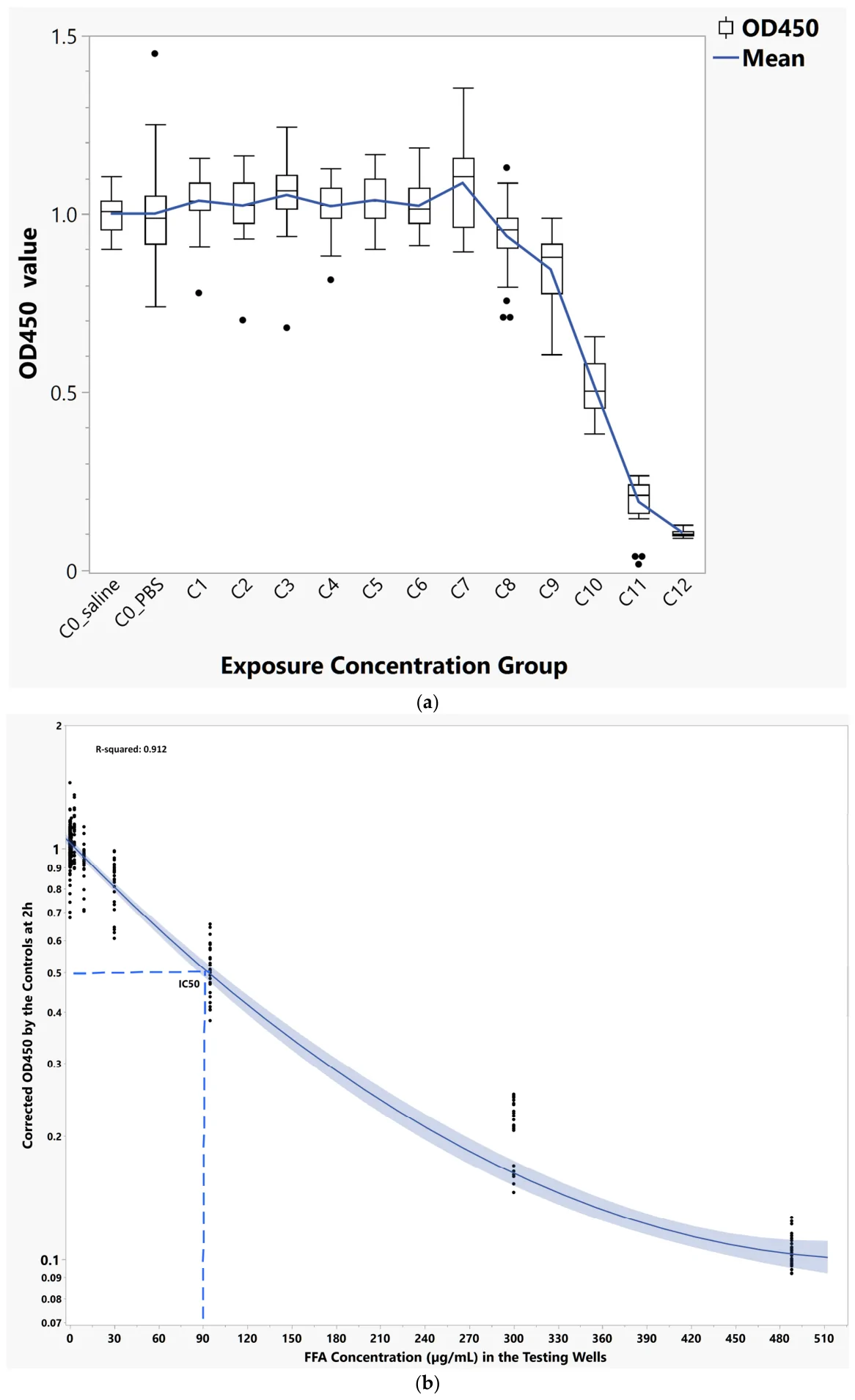
(**a**) Effects of FFA on the cultured human corneal epithelium cell (HCEC). HCECs were incubated for 48 h with various concentrations of FFA before the OD450 was measured using the CCK-8 assay. C0_saline = no FFA added in the culture medium and served as a control for tested concentrations (C1–C7) in which FFA was dissolved in saline; similarly, C0_PBS = no FFA added in the culture medium and served as a control for tested concentrations (C8–C12) in which FFA was dissolved in PBS (phosphate buffered saline). C1 = FFA 0.003 µg/mL (0.0094 µM), C2 = 0.00948 µg/mL (0.0297 µM), C3 = 0.03 µg/mL (0.0941 µM), C4 = 0.0948 µg/mL (0.2974 µM), C5 = 0.3 µg/mL (0.9412 µM), C6 = 0.948 µg/mL (2.9741 µM), C7 = 3 µg/mL (9.4118 µM), C8 = 9.48 µg/mL (29.7412 µM), C9 = 30 µg/mL (94.1176 µM), C10 = 94.8 µg/mL (297.4118 µM), C11 = 300 µg/mL (941.1765 µM), and C12 = 488 µg/mL (1530.9804 µM). The dots in the graph represent the outliers of the data points. (**b**) The graph of HCEC viability by the tested concentrations of FFA. The viability was represented by the OD450 readout from the testing wells, corrected by OD450 from the controls. The black dots show data points while the solid blue line show quadratic regression of the corrected OD450 values. The blue shaded area around the regression line represents the 95% confidence interval for the fitted mean. The FFA concentration needed to reduce the HCEC viability by half was about 90 µg/mL (IC50).

Human corneal epithelium cells (HCECs) were exposed to the stepped FFA concentrations (Figure 4a). The concentration of FFA that caused a 50% reduction in HCEC viability (IC50) was determined to be 90 µg/mL (282.4 µM) from a quadratic regression of the OD450 values on the testing concentrations (R^2^ = 0.912, Figure 4b).

### 3.3. FFA Permeation of Ocular Surface

**Figure 5:**
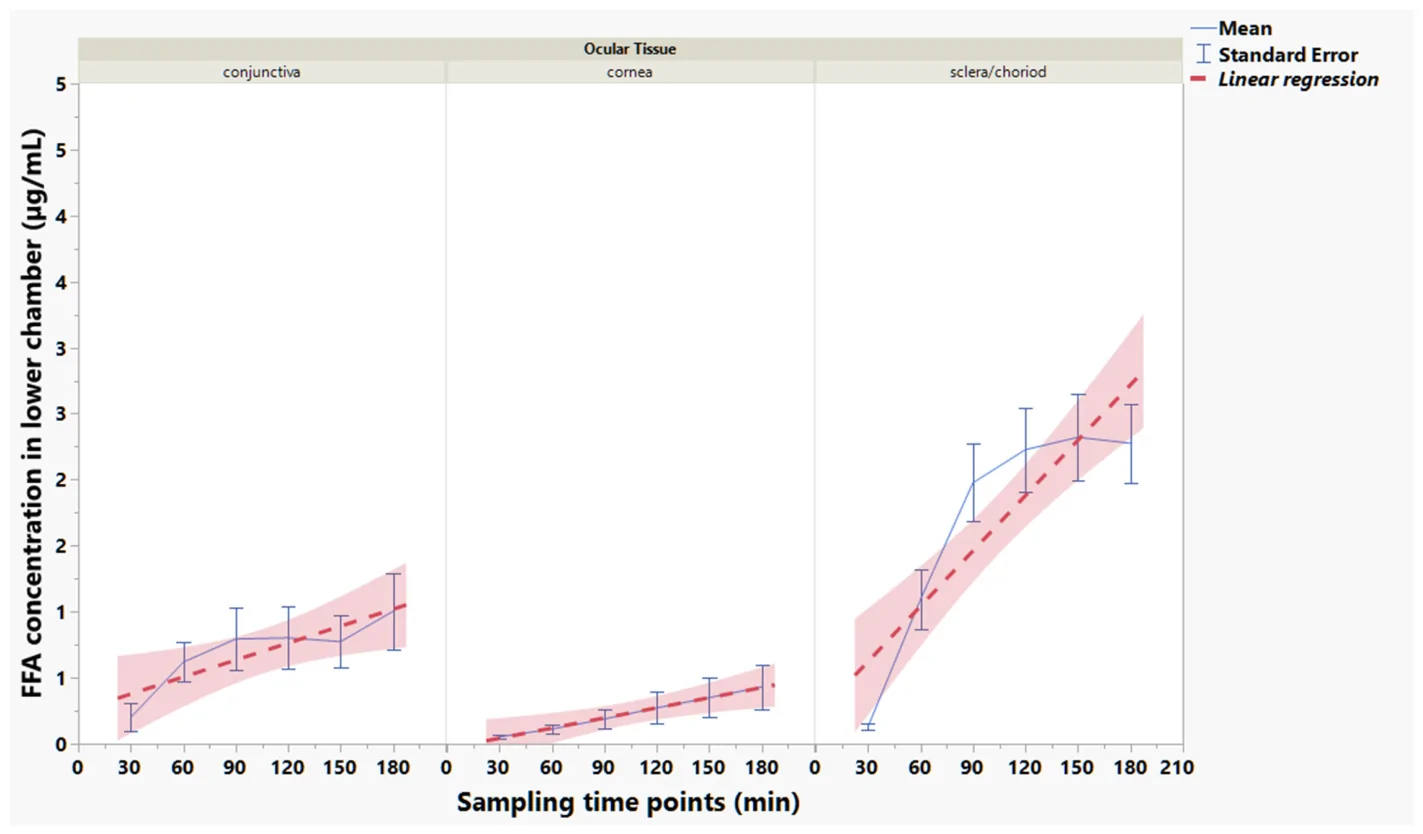
FFA permeation profiles through rat cornea, conjunctiva, and sclera/choroid complex. The data shows real-time concentration of the sample collected at the designated time after the pump starts.

It was clear that FFA penetrated the cornea, conjunctiva, and sclera (Figure 5). The FFA concentration in the lower chamber increased over time. The overall average FFA concentration under the cornea was 0.23 ± 0.32 (median 0.11) µg/mL, 0.70 ± 0.62 (median 0.74) under the conjunctiva, and 1.67 ± 1.33 (median 1.46) under the sclera/choroid. The average concentration in the chamber under the cornea is significantly lower than that under the conjunctiva and the complex of the sclera/choroid (Cornea vs. Conjunctiva, *p* < 0.0001; Cornea vs. Sclera/Choroid, *p* < 0.0001; Conjunctiva vs. Sclera/Choroid, *p* < 0.0001 Nonparametric Comparison for Each Pair Using Wilcoxon Method). The FFA permeation rates were calculated from the linear regression line between the sampling times 60 to 150 min. The permeation rate for the cornea was 1.77 × 10^−6^ µg/cm^2^/min, 4.95 × 10^−6^ µg/cm^2^/min for the conjunctiva, and 11.32 × 10^−6^ µg/cm^2^/min for the complex of the sclera/choroid.

### 3.4. Safety Profile After Topical Eyedrops of FFA

**Figure 6:**
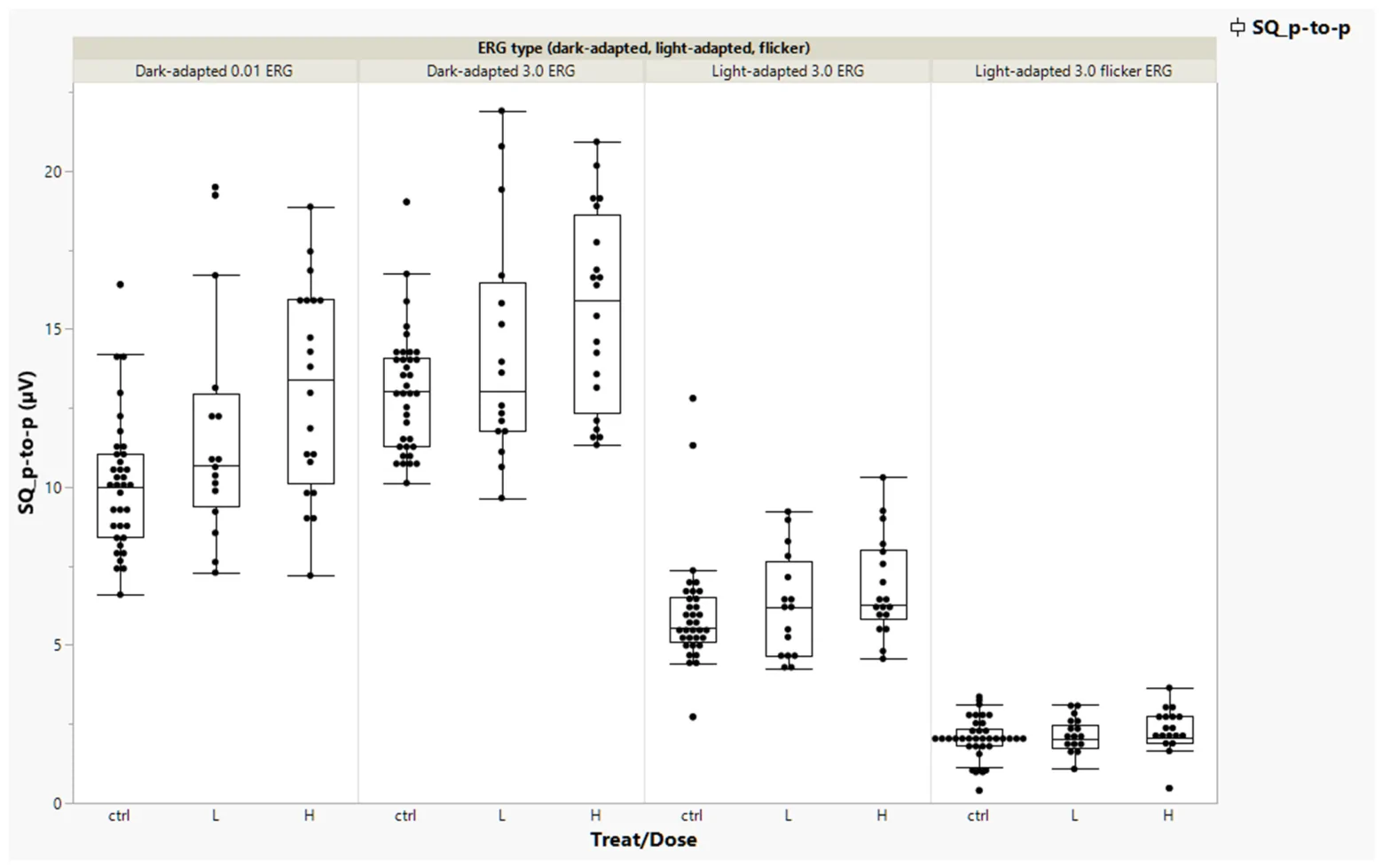
Square-root-transformed amplitude of the peak-to-peak (SQ_p-to-p) of ERGs from the eyes received twice daily saline (Ctrl), 90 µg/mL of FFA (L), or 500 µg/mL of FFA (H) eye drops for 2 weeks, stratified by the types of the ERGs.

The tested IC50 of FFA for HCEC was 90 µg/mL. In vitro testing was under a static condition. In contrast, an eye drop on a cornea is a dynamic situation in which the medication is diluted by the tear and washed away quickly after the application. Based on our experience, a 5- to 6-folds-higher concentration than the IC50 could be ideal for eyedrop application. Two concentrations, 90 µg/mL and 500 µg/mL, were tested for safety on the SD rat eyes. Four rats were used for the 90 µg/mL concentration and the other four rats for the 500 µg/mL concentration. The left eyes were used as controls. Both concentration solutions had a similar neutral pH value (≈7) to PBS, tested by a precision pH test strip (Shanghai Sanais Reagent Co., Ltd. Extensive PH test strip). No conjunctival congestion or cornea fluorescein staining was observed during the two-week application. The average body weight at the start of the experiment was 397.5 + 5.35 g, and the body weight increased by 26.88 g (*p* = 0.0002, paired *t*-test) to 424.4 ± 15.68 g two weeks later. ERG demonstrated similar peak-to-peak amplitudes from all three groups (control, low dose, and high dose) under the photopic ERG and the flicker ERG mode (Figure 6). For the dark-adapted ERG with either a light intensity of 0.01 cd.s/m^2^ or 3 cd.s/m^2^, the eyes with the high concentration of FFA eye drops showed a higher average peak-to-peak amplitude compared with the eyes with the saline eye drops (*p* = 0.0028 or *p* = 0.0054, the mixed model regression while adjusting for ERG performing time points, day 7 and day 14).

### 3.5. Ocular Pharmacokinetics Follow a Single Drop of 500 µg/mL of FFA

**Figure 7:**
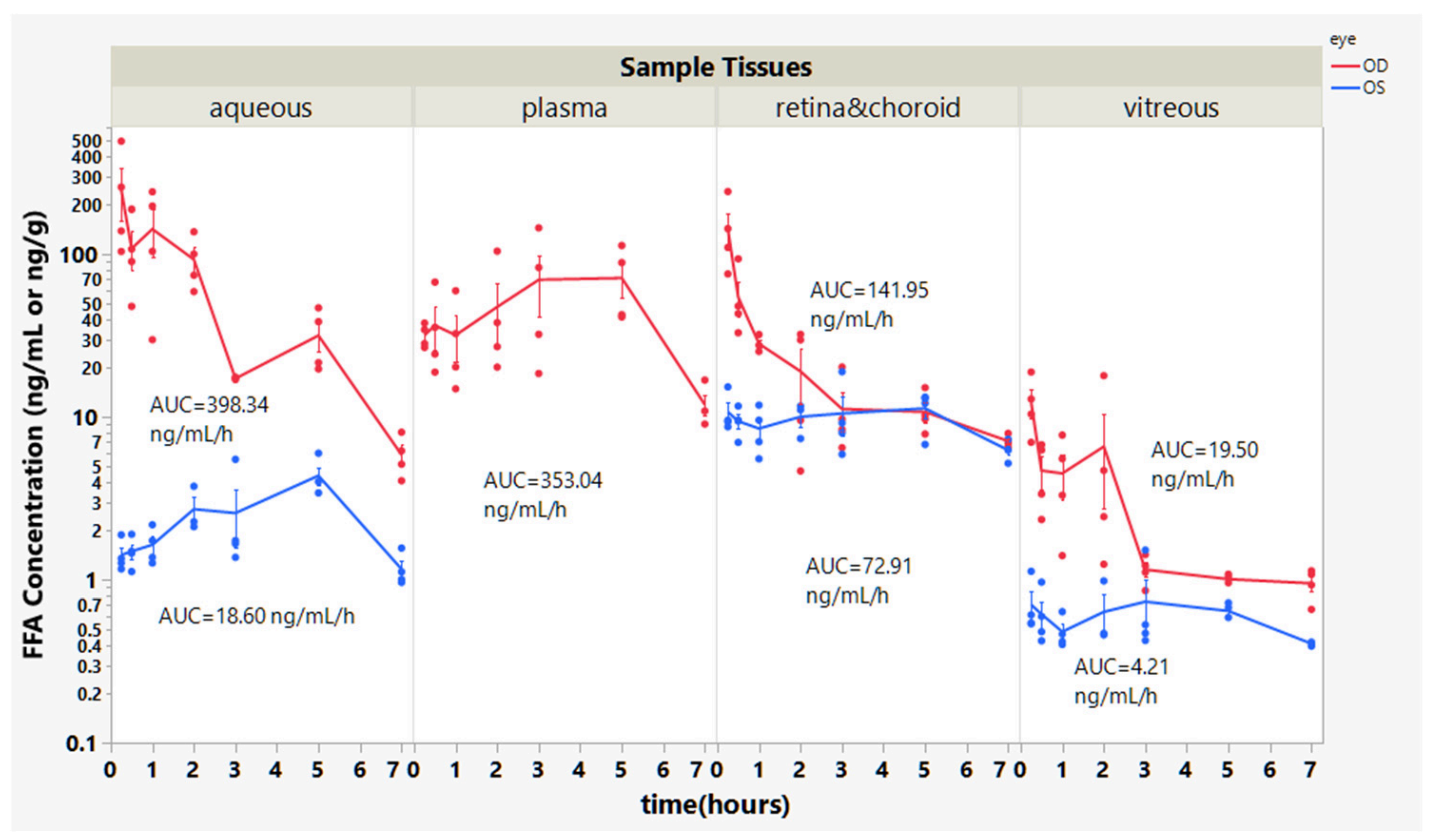
FFA concentration changes over time in the plasma and the ocular tissues following a single drop of FFA solution (500 µg/mL).

Based on the above safety study, FFA 500 µg/mL (0.05%) was used for the pharmacokinetics study. The justification is that we did not observe any ocular surface issues or weight loss in the animals. In addition, the amplitudes of the dark-adapted ERG were higher, not lower. The FFA concentration profiles in the various ocular tissues and in the plasma were presented in Figure 7. FFA was also detected in the contralateral eyes. However, drug exposure to the contralateral eye aqueous humor was only 5% of the exposure to the study eye aqueous humor. In contrast, FFA systemic exposure was close to that of the study eye aqueous humor, and FFA exposure to the retina/choroid of the contralateral eyes reached about 50% of that to the study eyes (Figure 7). FFA peak concentration was highest in the aqueous humor (Cmax, mean maximum concentration, = 249 ± 88 <SE> ng/mL), followed by the retina/choroid (Cmax = 143 ± 36 <SE> ng/g), and then the vitreous (12 ± 3 <SE> ng/mL). FFA peak concentration was delayed by 3 to 4 h (Cmax for the plasma was 72 ± 17 <SE> ng/mL). After one drop of FFA 500 µg/mL, FFA was detectable in ocular tissues and in plasma for at least 7 h.

### 3.6. Efficacy of FFA Eye Drops (500 µg/mL) on BAC-Induced Dry Eye Model in SD Rats

**Figure 8:**
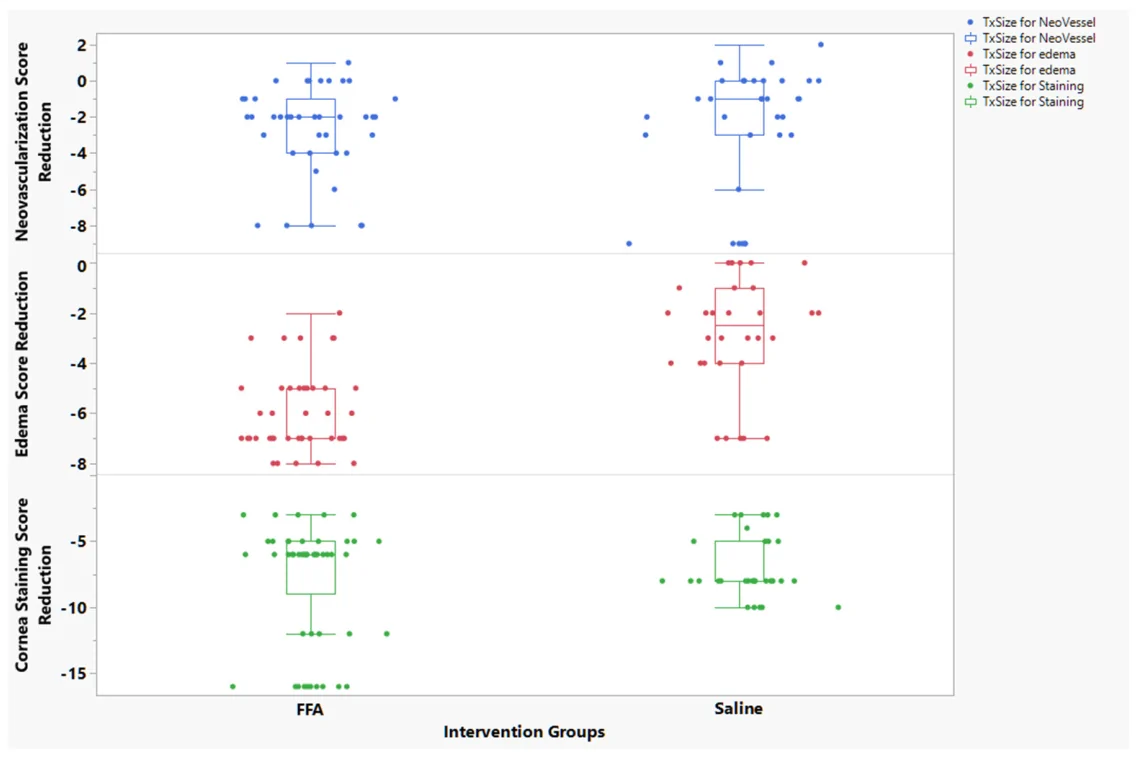
Box-plots of the corneal score reduction from the intervention, stratified by corneal pathologies and the intervention groups.

With the regimen of 0.2% BAC drops 3 times a day, the cornea fluorescein staining reached the pre-set criteria to initiate the intervention within an average time of 5.76 ± 2.56 days. The reduction in the scores for corneal neovascularization, corneal edema, and corneal staining during the treatment course were presented in Figure 8. Compared with eyes treated by saline drops, the eyes treated by FFA drops had a significantly lower median score reduction for corneal neovascularization and corneal edema. The median scores of the reduction for cornea neovascularization and cornea edema were −2 and −7 for FFA eyes, while it was only −1 and −2.5 for saline eyes (*p* = 0.0294, *p* < 0.0001, median tests). For fluorescein staining, the rat corneal epithelium had a fast recovery, and not much corneal fluorescein staining remained on the first slit-lamp examination (day 4 of the treatment course). No significant difference in cornea staining was present between the eyes with FFA and saline. Compared with the cornea staining, cornea edema and neovascularization were lingering during the treatment course.

**Figure 9:**
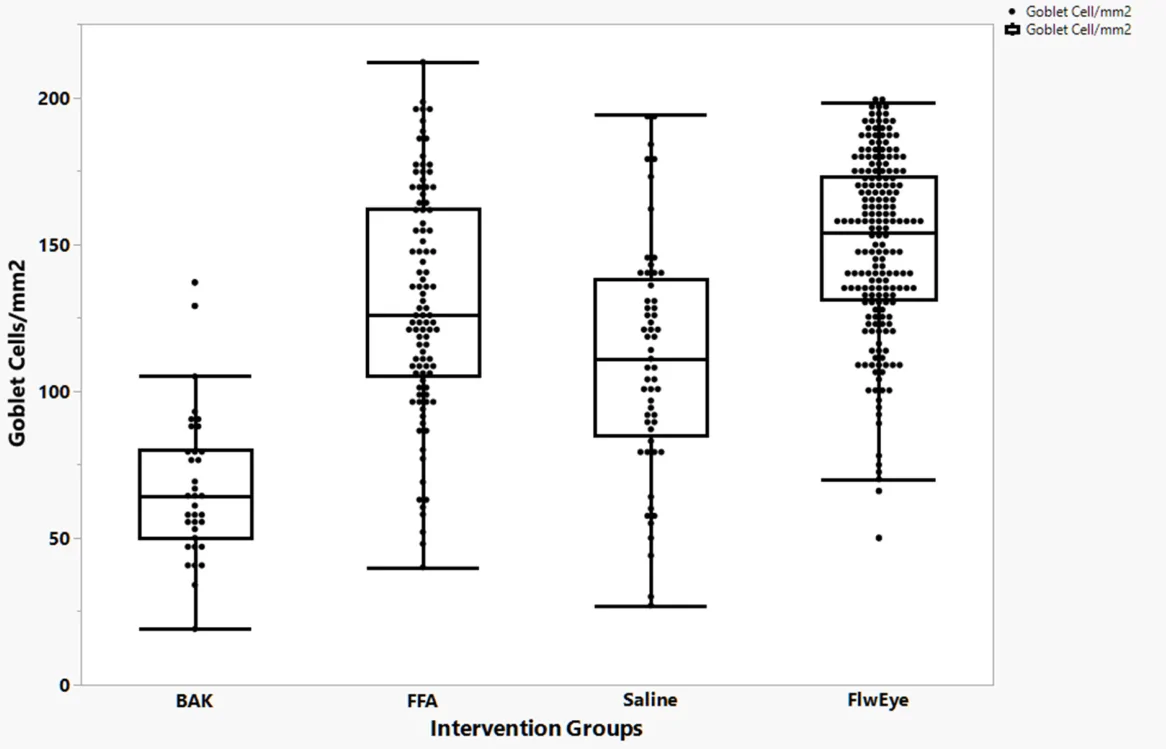
Box-plots of the goblet cell counts from conjunctival impression cytology, stratified by the intervention groups.

Goblet cell counts from the conjunctival impression cytology were presented in Figure 9 for pre-intervention (BAC), interventions, and the fellow eyes. The analysis revealed that both FFA and saline drops promoted significant goblet cell recovery; however, the goblet cell numbers in the eyes with FFA drops was significantly higher than those of the saline eyes (FFA vs. Saline, 20 cells/mm^2^ more for the eyes with FFA drops, 32 to 8 95% confidence interval, *p* = 0.0014). Though goblet cells had a significant recovery from BAC toxicity with FFA drops, the mean cell number did not reach the level of the fellow eyes without BAC exposure (19 cells/mm^2^ less than that of the fellow eyes, 27 to 10 95% confidence interval, *p* < 0.0001).

## 4. Discussion

FFA is a PPAR-alpha agonist and inhibits inflammation by multiple pathways such as suppressing inflammation-related gene expression and modulating T-cell differentiation, in addition to activating the PPAR-alpha pathway [14]. Fenofibrate or fenofibric acid have been experimentally formulated into eye drops and can reach the retina of rodent eyes for therapeutic potential for retinal inflammatory diseases [8,21]. The current study is the first to investigate the potentials of topical FFA on a dry eye model. An in vitro dissolution study demonstrated that FFA can be dissolved in PBS much more than in saline. The PBS buffer capacity not only led to a higher FFA concentration but also maintained the pH at neutral for topical eye application, which allowed us to formulate FFA eye drops to be five to six times higher than the IC50 to inhibit the cultured human corneal epithelium cell line. Eye drops are often formulated to have higher concentrations than the IC50 from in vitro cytotoxicity [22]. Taking diclofenac as an example, a 0.1% ophthalmic solution (preservative-free) is 10 to 15 times higher than the IC50 on corneoconjunctival epithelial cell lines [23]. The underlying consideration is that topically applied eye drops have a much shorter duration of exposure on the eye surface. The current study had a concentration of FFA of 0.05% for topical application with a pH of 7 and an osmolarity of 293.1 mOsm/L. Though the FFA solution was targeted to be a topical application, the ability to penetrate the ocular surface layers may be closely associated with its safety and the duration of the therapeutics’ retention. The current study revealed that the FFA permeation rate through the conjunctiva was greater than that of the cornea. This may very likely be associated with the thickness and tighter arrangement of epithelial cells. The corneal thickness of SD rats is about 180 µm, with one-third of the thickness consisting of the epithelium, while the conjunctiva has only 50 µm on average [24]. The good penetration of the conjunctiva is an advantage for FFA to quench the conjunctival inflammation, which has a rich distribution of mucin-producing goblet cells and is considered a key contribution to dry eye formation. In addition, the sclera/choroid complex demonstrated a good permeation of FFA. This may facilitate the topical FFA to reach the retina in rodent eye models [8,21].

The FFA topical solution in the current study was neutral pH and isotonic. No observational cornea fluorescein staining or conjunctival congestion was found. Systemic body weight monitoring did not reveal any abnormality, either. Repeated ERG examinations demonstrated similar amplitudes to the control eyes for light-adapted ERG and 30 Hz flicker ERG, which is the most sensitive test to reveal subtle and early changes for ocular toxicity. Scotopic ERGs revealed that the eyes with high-concentration FFA eye drops demonstrated higher mean ERG amplitudes when compared with the control eyes or the eyes with low-concentration FFA. The peak-to-peak amplitude was higher instead of lower; it is possible that visual rod cells keep a more responsive status to light due to the FFA effect. However, the real underlying cause cannot be elucidated from the current study, and such an observation may not be consistent. More studies from different study groups would shed further light on this observation. Nonetheless, the other safety measures did not raise concerns; especially if used on the human eye, the chance of FFA reaching the retina after topical application may be very limited.

The pharmacokinetics of FFA in ocular tissues after a single drop of application revealed interesting findings. It is clear that FFA in the study eyes (OD) was significantly higher than that in the control eyes (contralateral eyes), which can be seen from the study eyes’ AUC being 21 times higher than that of the contralateral eyes. For the retina/choroid, FFA was higher in the study eyes at the early time points, but not much difference could be appreciated at the later time points between the study and the contralateral eyes. This may be explained by the high AUC of the plasma. The AUC of the plasma was even higher than the AUC of the study eyes. It seems that topical FFA was well-absorbed into the systemic circulation, and FFA was accumulated in the retina and choroid due to their rich blood supplies. It is very likely that the high AUC of the plasma is due to the small body weight of rodents, which indicates that rodents are not ideal models for testing topical eye drops if the study purpose is targeting retinal diseases. In the case of human eyes, topically absorbed medication would distribute among a much larger body weight, leading to a low concentration in systemic circulation.

Benzalkonium chloride (BAC)-induced rodent ocular surface toxicity has been well-characterized and used as a type of experimental dry eye model [25,26]. Macroscopic and slit-lamp examinations revealed corneal epithelial disruption with fluorescein staining, corneal edema, and corneal neovascularization, along with microscopic evidence of the loss of conjunctival goblet cells from the impression cytology, which have been the reliable outcome measures for ocular surface damages in this rodent model. The current study demonstrated that FFA drops promoted the regression of cornea edema and neovascularization as compared with saline eye drops. The current study used Sprague–Dawley rats as the model. The rat cornea was quickly re-epithelialized once BAC was discontinued. Corneal gross fluorescent staining disappeared on the first slit-lamp examination, which was on day 4 of FFA or saline intervention. Different animals or even different strains of the same animal may differ in their resistance to BAC epithelial toxicity [26]. In the current study, a prominent manifestation of cornea toxicity to BAC was the cornea edema or opacity. The cornea opacity was easily identified under a slit-lamp examination, and the opacity takes time to resolve even upon the FFA intervention. Therefore, cornea opacity could be a reliable outcome measure for the quantitation of the therapeutic efficacy of topical eye drops on this dry eye model. Another sensitive measure of this SD dry eye model is the conjunctival impression cytology, the number of goblet cells as a hallmark of dry eye pathology. The number of conjunctival goblet cells reduced significantly (by 55%) on BAC drops, which can be seen by comparing the goblet cell number of BAC-applied eyes prior to the intervention with the number of their fellow eyes. After the initiation of the intervention, the goblet cell recovery was significantly greater from the FFA intervention than that from saline drops; however, the recovery did not reach the levels of the fellow eyes during the treatment course. Nonetheless, comparing saline with FFA, FFA drops promote goblet cell recovery by 20 cells per mm^2^ of the counting field, or 18% over saline eye drops.

In conclusion, the current study demonstrated that a 0.05% FFA eye drop is safe for topical application on this rat dry eye model. The ocular pharmacokinetics indicated that the eye drop is the appropriate form factor to target ocular surface inflammation. Twice-a-day application of the FFA eye drops demonstrated a significant benefit of corneal edema resolve and a significant protective effect against the loss of the conjunctival goblet cells. This novel finding shone light on a new perspective to develop a new treatment and advance the treatment for dry eyes.

## Data Availability

The data that support the findings of this study are available from the corresponding author [Cheng L.] upon reasonable request.
